# Quantity of Carbonic Acid Exhaled by Man in Health and Disease

**Published:** 1849-06

**Authors:** 


*Quantity of Carbonic Acid exhaled by Man in Health and Disease.*—We extract the following from a Memoir on the Variations of Carbonic Acid exhaled in a state of disease and in certain physiological conditions, such as sleep, digestion, etc., lately presented to the Academy of Sciences:

*Physiological State.*—1st. There exists in the exhalation of carbonic acid hourly variations corresponding with those of the barometer, having as the latter, two maximums, the one about 9 o'clock in the morning, and the other at 11 o'clock in the evening; and two minimums, the one about 3 P.M., and the other at 5 A.M. The maximum of the morning is greater than that of the evening.

2d. Variations of temperature and pressure act in an inverse ratio of one another; the one to diminish the other to augment the exhalation of carbonic acid gas.

3d. During the process of digestion there is less carbon burnt.

4th. Animal food diminishes, while the exclusive use of feculent matter augments, the quantity of carbonic acid.

5th. During rapid exercise, the air expired does not contain more carbonic acid.

6th. The same is true after breathing ether and chloroform.

7th. The use of alcoholic drinks produces the same effect.

8th. Less carbonic acid is generated during sleep than during wakefulness.

9th. The temperature of the air expired in a state of health does not sensibly vary.

10th. The air expired by infants contains more carbonic acid than that of adults.

*Pathological State.*—1st. In meningitis, peritonitis, metro-ovaritis, and generally in all well characterized phlegmasia, there is carbonic hypercrinia.

2d. The exceptions to this rule are: pneumonia, pleurisy, pericarditis, and all the phlegmasia which produce embarrassment of the respiration or circulation. In this case, there is carbonic hypocrinia.

3d. Subjects laboring under acute articular rheumatism exhale more carbonic acid.

4th. More carbon is burnt during a paroxysm of intermittent fever. The increase is more marked during the hot stage than during the chill. Towards the close of the sweating stage, the air expired differs but slightly from that expired in a state of health.

5th. In all diseases which are not accompanied by fever or marasmus, as chlorosis, diabetes, and nervous affections, there are no variations in the properties of carbonic acid expired.

6th. In variola, rubeola, roseola, scarlatina, erysipelas, and erythema, there is less carbon consumed.

7th. During the work of suppuration, the lung exhales less carbonic acid.

8th. In scurvy, purpura, anemia, and anasarca, there is carbonic hypocrinia.

9th. The same is the case in the last stages of the cancerous, scrofulous and syphilitic cachexia.

10th. Individuals affected with typhoid fever, chronic dysentery or diarrhea, exhale less carbonic acid.

11th. There is less carbon burnt by the respiration in pulmonary phthisis.

12th. The temperature of the air expired in disease is in direct ratio to the number of inspirations.—*Gazette des Hôpitaux.*—D. W. Y.

